# The PAPI-1 pathogenicity island-encoded small RNA PesA influences *Pseudomonas aeruginosa* virulence and modulates pyocin S3 production

**DOI:** 10.1371/journal.pone.0180386

**Published:** 2017-06-30

**Authors:** Silvia Ferrara, Marilena Falcone, Raffaella Macchi, Alessandra Bragonzi, Daniela Girelli, Lisa Cariani, Cristina Cigana, Giovanni Bertoni

**Affiliations:** 1 Department of Biosciences, Università degli Studi di Milano, Milano, Italy; 2 Infections and Cystic Fibrosis Unit, Division of Immunology, Transplantation and Infectious Diseases, IRCCS San Raffaele Scientific Institute, Milano, Italy; 3 Cystic Fibrosis Microbiology Laboratory, Fondazione IRCCS Ca' Granda, Ospedale Maggiore Policlinico, Milano, Italy; Centre National de la Recherche Scientifique, Aix-Marseille Université, FRANCE

## Abstract

Small non-coding RNAs (sRNAs) are post-transcriptional regulators of gene expression that have been recognized as key contributors to bacterial virulence and pathogenic mechanisms. In this study, we characterized the sRNA PesA of the opportunistic human pathogen *Pseudomonas aeruginosa*. We show that PesA, which is transcribed within the pathogenicity island PAPI-1 of *P*. *aeruginosa* strain PA14, contributes to *P*. *aeruginosa* PA14 virulence. In fact, *pesA* gene deletion resulted in a less pathogenic strain, showing higher survival of cystic fibrosis human bronchial epithelial cells after infection. Moreover, we show that PesA influences positively the expression of pyocin S3 whose genetic *locus* comprises two structural genes, *pyoS3A* and *pyoS3I*, encoding the killing S3A and the immunity S3I proteins, respectively. Interestingly, the deletion of *pesA* gene results in increased sensitivity to UV irradiation and to the fluoroquinolone antibiotic ciprofloxacin. The degree of UV sensitivity displayed by the PA14 strain lacking PesA is comparable to that of a strain deleted for *pyoS3A*-*I*. These results suggest an involvement of pyocin S3 in DNA damage repair and a regulatory role of PesA on this function.

## Introduction

Bacterial small RNAs (sRNAs) have been recognized as key contributors to regulatory networks, and have been shown to play critical roles in many intra- and extracellular processes, and in pathogenesis [1–3]. Most sRNAs exert their regulatory function post-transcriptionally, acting by base pairing with the mRNA of their target genes ultimately modulating mRNA translation and/or stability. sRNAs can share extended base complementarity when they are *cis*-encoded on the opposite strand of the target mRNA, or they can interact with the target mRNA via short and imperfect base pairing, as in the case of *trans*-encoded sRNAs. The expression of most sRNAs is responsive to environmental stress conditions spanning from iron and oxygen limitation, to oxidative, metabolic/nutrient, pH and cell envelope stresses [4].

In the opportunistic human pathogen *Pseudomonas aeruginosa* the use of transcriptomics approaches has recently led to the identification of numerous new sRNAs, mostly in the attenuated strain PAO1 and some also in the virulent one PA14 [5–7]. The bacterium *P*. *aeruginosa* is known as a major cause of both acute and chronic lung infections in patients belonging to all age groups who are immunocompromised, or who have defective mucociliary clearance, previous epithelial injury or foreign body placement [8]. Lung infections caused by *P*. *aeruginosa* can appear as a spectrum of clinical entities ranging from a rapidly fatal pneumonia in a neutropenic patient to a multi-decade bronchitis in patients with cystic fibrosis (CF). The expression of virulence traits in *P*. *aeruginosa* is fine-tuned by a dynamic and intricate regulatory network [9],that leads the expression of *P*. *aeruginosa* pathogenic functions with a sharp timing. In this scenario, sRNAs can finely contribute to *P*. *aeruginosa* ability to quickly adapt to a new environment and manage to persist.

Previously, bioinformatics and approaches such as the pull-down with the RNA chaperone Hfq have been used for identifying novel *P*. *aeruginosa* sRNAs but only a small number of them have been characterized functionally, and even less described as being implicated in the regulation of *P*. *aeruginosa* virulence [10–17]. Moreover, most of the studies have been performed using the reference strain PAO1, while very little is known about the biological effects of the sRNA mediated regulation in virulent *P*. *aeruginosa* strains, such as PA14. Compared to PAO1, the clinical isolate PA14 is significantly more virulent in a wide range of hosts, including mice, the nematode *Caenorhabditis elegans*, the insect *Galleria mellonella*, and the plant *Arabidopsis thaliana*, and has thus become an important reference strain because of its enhanced virulence [18–20]. Generally, PAO1 and PA14 strains share highly conserved genomes, although PA14 possesses a slightly larger one, likely due to horizontal gene transfer resulting in the acquisition of pathogenicity islands (PAIs) [21].

This study focuses on a *P*. *aeruginosa* sRNA observed for the first time by a sRNA-sequencing approach [5], in which unique and conserved sRNAs in the *P*. *aeruginosa* strains PAO1 and PA14 were revealed. Here, we validated the sRNA with the operative name SPA0021, renamed PesA, as being transcribed from the pathogenicity island PAPI-1, present in PA14 strain, but not in PAO1. In addition, our results display that PesA is expressed in several *P*. *aeruginosa* isolates, including environmental and clinical ones isolated from CF patients. Moreover, we show that PesA operates a post-transcriptional regulation of genes involved in S-type pyocin production. Pyocins are narrow-spectrum bacteriocins synthesized by more than 90% of *P*. *aeruginosa* strains and presumed to play a role in niche establishment and protection in mixed populations. The pyocin genes are usually located on the *P*. *aeruginosa* chromosome and induced by mutagenic agents that cause DNA damage such as mitomycin C and UV irradiations. *P*. *aeruginosa* pyocins can be subdivided into insoluble R and F pyocins and soluble S pyocins. S-type pyocins AP41, S1, S2, and S3 are constituted of two components in which the large component carries an endonuclease C-terminal domain responsible for the killing activity that causes cell death by DNA cleavage [22–27], while pyocins S4 and S5 have tRNAse and pore-forming activities, respectively [28, 29]. More recently, also the new S-type pyocin S6, with rRNase activity, has been described functionally [30]. In our study, we show that PesA deletion leads to decreased expression of pyocin S3, associated to increased sensitivity to UV irradiation and to the fluoroquinolone antibiotic ciprofloxacin. Furthermore, PesA is induced by host-environment stimuli such as low oxygen availability and body temperature, suggesting a key role in *P*. *aeruginosa* adaptability to different environmental stresses. Finally, we also found that PesA is involved in the regulation of *P*. *aeruginosa* PA14 virulence in CF human bronchial epithelial cells. Our results suggest that a PAPI-1-encoded sRNA can contribute to the modulation of the expression of genes outside PAPI-1, and to different aspects of *P*. *aeruginosa* pathogenesis during infection.

## Materials and methods

### Ethics Statement

Study on human *P*. *aeruginosa* isolates from Hannover has been approved by the Ethics Commission of Hannover Medical School, Germany [31]. The patients and parents gave oral informed consent before the sample collection. Approval for storing of biological materials was obtained by the Ethics Commission of Hannover Medical School, Germany. The study on human *P*. *aeruginosa* isolates from the Regional CF Center of Lombardia (S1 Table) was approved by the Ethical Committees of San Raffaele Scientific Institute and Fondazione IRCCS Ca’ Granda, Ospedale Maggiore Policlinico, Milan, Italy, and written informed consent was obtained from patients enrolled or their parents according to the Ethical Committees rules, in accordance with the laws of the Italian Ministero della Salute (approval #1874/12 and 1084/14).

### Bacterial strains and culture conditions

Bacterial strains and plasmids used in this study are listed in S2 Table. *E*. *coli* strains were routinely grown in Luria-Bertani broth (LB) at 37°C. *P*. *aeruginosa* strains were grown at 37°C in Brain Heart Infusion (BHI) rich medium or in LB at 120 rpm unless otherwise indicated. Carbenicillin and gentamicin were added at 300 and 20 μg/ml, respectively, unless otherwise indicated. For *P*_*BAD*_ induction in vector plasmid pGM931, arabinose was added to a final concentration of 10 mM. Anaerobic batch cultivations of *P*. *aeruginosa* and the shift from aerobic to anaerobic conditions were performed in a 800 ml-Biostat-Q system bioreactor (B-Braun) as described previously [17].

### Plasmid constructions and mutant generations

Oligonucleotides used in this study are listed in S3 Table. To construct plasmid pGM-*pesA*, the *pesA* gene was PCR-amplified from PA14 genomic DNA with oligos 9/10, digested with *Nco*I-*Pst*I and cloned into the pHERD20T derivative vector pGM931 carrying a transcriptional terminator downstream the multicloning site [32, 33].

Plasmids pBBR1-*pyoS3I*::*sfGFP*, pBBR1-*leader-pyoS3A*::*sfGFP*, pBBR1-*lacZ*::*pyoS3A-I*::*sfGFP*, and pBBR1-*mCherry*::*pyoS3A-I*::*sfGFP* expressing *pyoS3I*::*sfGFP*, *pyoS3A*::*sfGFP*, *lacZ*::*pyos3A-I*::*sfGFP*, or *mCherry*::*pyos3A-I*::*sfGFP* translational fusions, respectively under the *P*_*LtetO-1*_ constitutive promoter were constructed as follows. A DNA fragment including the last 39 codons of the open reading frame of Pyos3A and 36 first codons of PyoS3I was amplified by PCR with oligos 11/12, digested with *Nsi*I-*Nhe*I and cloned into the sfGFP reporter vectors pXG10-SF and pXG30-SF [34] giving rise to plasmid pXG10-*pyoS3I*::*sfGFP* and pXG30-*lacZ*::*pyoS3A-I*::*sfGFP*, respectively. A DNA fragment including the 278-nt UTR and the first 37 codons of the *pyos3A* open reading frame was amplified with oligos 13/14 digested with *Nsi*I-*Nhe*I and cloned into the sfGFP reporter vectors pXG10-SF giving rise to plasmid pXG10-*leader-pyoS3A*::*sfGFP*.

The DNA fragments spanning from the *P*_*LtetO-1*_ promoter to the end of the GFP reporter gene were amplified by PCR respectively from pXG10-*pyo-S3I*::*sfGFP*, pXG30-*lacZ*::*pyoS3A-I*::*sfGFP* and pXG10-*leader-pyoS3A*::*sfGFP* with oligos 18/19, digested with *Cla*I-*Xba*I and cloned into the low-copy number shuttle vector pBBR1-MCS5 giving rise to constructs pBBR1-*pyoS3I*::*sfGFP*, pBBR1-*lacZ*::*pyoS3A-I*::*sfGFP* and pBBR1-*leader-pyoS3A*::*sfGFP*, respectively. mCherry gene was amplified by PCR from the pMMR plasmid [35] using primers 15/16, (in which forward primer contained the Shine-Dalgarno sequence, and reverse primer lacked mCherry stop codon sequence), digested with *NsiI* and cloned into the pXG10-*pyoS3I*::*sfGFP* previously digested with *Nsi*I, giving rise to plasmid pXG10-*mCherry*::*pyoS3A-I*::*sfGFP*. The DNA fragment spanning from the *P*_*LtetO-1*_ promoter to the end of the GFP reporter gene was amplified by PCR using oligos 18/19 digested with *Cla*I-*Xba*I and cloned in the pBBR1-MCS5 vector, giving rise to plasmid pBBR1-*mCherry*::*pyoS3A-I*::*sfGFP*. Plasmid pBBR1-*mCherry* expressing mCherry reporter gene under *P*_*LtetO-1*_ was constructed as follows. The DNA fragment including the *P*_*LtetO-1*_ promoter and the mCherry reporter gene was amplified from pBBR1-*mCherry*::*pyoS3A-I*::*sfGFP* using oligos 17/18 (in which reverse primer contained *mCherry* stop codon). The PCR product was digested with *Cla*I-*Xba*I and cloned in the pBBR1-MCS5 vector.

*P*. *aeruginosa* mutant in *pesA* gene was generated by an enhanced method of markeless gene replacement described previously [36] with some modifications to adapt it to *P*. *aeruginosa* as described previuosly [17]. PA14 mutant in *pesA* gene was obtained by allelic exchange with a deletion from—34 to + 140 positions with respect to *pesA* transcription start site as follows. TS1 region spanning left 533 bp flanking sequence of *pesA gene* was amplified by PCR with oligos 23/24. TS2 region spanning last 127 nt and right 368 bp flanking sequence of *pesA* was amplified by PCR with oligos 25/26. PCR amplifications were performed from PA14 genomic DNA. Overlap extension (SOE)-PCR with oligos 23/26 was used to join TS1 and TS2 that carried end complementary regions introduced by 25/26, respectively during their separate PCR amplification [37]. Joined TS1-TS2 DNA fragments were digested with *Eco*RI-*Pst*I and cloned in CC118 λ*pir* into the poly-linker site of pSEVA612S giving rise to pSEVApa14-*ΔpesA*.

The TS1-TS2–inserted pSEVApa14-*ΔpesA* was transferred from *E*. *coli* CC118 λ*pir* to PA14, with the assistance of the helper *E*. *coli* strain HB101(pRK600) in a conjugative triparental mating. Exconjugant *P*. *aeruginosa* clones were selected on M9-citrate with 60 μg ml^−1^ of gentamicin. Since pSEVA612S derivatives cannot replicate in *P*. *aeruginosa*, Gm^R^ exconjugant clones could appear only by co-integration of the construct in the genome of the recipient strain by homologous recombination between joined TS1-TS2 fragments borne by pSEVA612S and the recipient chromosome. Plasmid pSW-1 was transferred from *E*. *coli* DH5α to *P*. *aeruginosa* clones bearing genomic co-integrates of pSEVApa14-*ΔpesA* by triparental mating as above, and pSW-1-recipient *P*. *aeruginosa* clones were selected on M9-citrate with 300 μg ml^−1^ of carbenicillin. Cultures of resulting *P*. *aeruginosa* clones carrying pSW-I were grown overnight in LB with 300 μg ml^−1^ of carbenicillin and then plated on the same medium. Single colonies were screened for loss of gentamicin resistance. Gentamicin-sensitive clones carrying the deleted alleles were then screened by PCR with oligo pairs 27/28 for *pesA*.

All plasmid constructs and deletion mutant were checked by sequencing with oligos indicated in S3 Table.

### RNA isolation and analysis

Total RNA was prepared as described previously [5] from 2–10 ml of bacterial cell cultures. Quality and concentration of the RNA extracted were assessed by a Biospectrometer (Eppendorf). Northern blot analyses were performed as described previously [5]. DNA oligonucleotide probes were 5'-end labeled with [γ-^32^P]ATP (PerkinElmer, NEG502A) and T4 polynucleotide kinase (Promega, M4103) according to manufacturer’s instruction. Oligo 1 and 2 were used to probe PesA and 5S RNA, respectively. Treatment with terminator-5’-phosphate-dependent exonuclease was performed in terminator reaction buffer A (Epicentre, TER51020) as described in detail previously [17].

Radioactive bands were acquired after exposure to phosphor screens using a Typhoon^™^ 8600 variable mode Imager scanner (GE Healthcare BioSciences) and visualized with imageQuant software (Molecular Dynamics).

Quantitative RT-PCR analysis was performed on total RNA extracted from *P*. *aeruginosa* PA14 wild-type and Δ*pesA* at mid-, late-exponential and stationary phase (OD_600_ of 0.8, 1.6 and 2.7, respectively). cDNA synthesis was performed from 1 μg of total purified RNA using QuantiTect Reverse Transcription Kit (Qiagen). RT-PCRs were performed using QuantiTect SYBR^®^ Green PCR Kit (Qiagen) and oligo pairs 30/31, 32/33, 34/35 for 16S, *pyoS3A* and *pyoS3I* amplification, respectively. The reaction procedure involved incubation at 95°C for 15s and 40 cycles of amplification at 94°C for 15 s, 60°C for 30 s and 72°C for 30s. 16S ribosomal RNA was used as reference.

### *In vitro* and *vivo* assays of sRNA/mRNA interactions

Electrophoretic Mobility Shift Assay (EMSA) to analyze sRNA/mRNA interactions were performed as described previously [17].

Experiments with fluorescent reporters were carried out as described previously [17]. Fluorescence polarization FP_485/535_, fluorescence intensity FI_590/635_ and Abs_595_ were measured in a Tecan Infinity PRO 200 reader, using Magellan as data analysis software (Tecan). GFP and mCherry activities were expressed in Arbitrary Units (AU) as ratio FP_485/535_/Abs_595_ and FI_590/635_/Abs_595_, respectively.

### RNA synthesis

RNAs for RNA/RNA interaction assays were prepared by T7 RNA polymerase transcription of gel-purified DNA fragments obtained by PCR as described previously [17]. DNA fragments for PesA RNA and *pyoS3A-I* mRNA preparations were amplified from *P*. *aeruginosa* PA14 genomic DNA with oligo pairs 3/4 and 5/6, respectively. The DNA fragment for RseX RNA was amplified from *E*. *coli* C1a genomic DNA with oligos 7/8.

### Pyocin S3 spotting assay

Pyocin killing assay was performed using the spotting method as indicated previously [38] with some modifications. 10 μl of filter-sterilized supernatants from cell cultures with OD_600_ of 1 were spotted onto LB 1.5% agar plates. A lawn of the pyocin S3 sensitive *P*. *aeruginosa* strain ATCC 27853 containing 5 × 10^6^ cells ml^−1^ was plated by inclusion into 0.3% soft agar over the dried spots. Plates were incubated overnight at 37°C and checked for the formation of the clearing zone on the spotting site, which is indicative of the pyocin S3 activity.

### Antibiotic disk diffusion

Susceptibilities of *P*. *aeruginosa* strains to antimicrobial agents were analyzed by disk diffusion measurement. Filter disks (Oxoid, CT0425B, CT0013B, CT0207B, CT0052B, CT0058, CT0010B) were placed on a lawn of 10^6^ CFU/ml bacterial cells plated by inclusion into 0.3% LB agar. Plates were incubated overnight at 37°C and the diameters of the clear zones around the disks were measured.

### UV sensitivity assay

UV treatment was performed using a Stratalinker 1800 UV Crosslinker (Stratagene). Cell cultures with an OD_600_ of 1 (corresponding to 8 x 10^8^ CFU/ml) were serially diluted until 10^−7^; 3 μl of each dilution were spotted in triplicate onto LB-agar plates, dried, and exposed to increasing amounts of UV radiation, from 0 to 100 J/m^2^. Plates were incubated overnight at 37°C.

### Cytotoxicity assays in human CF respiratory cells

IB3-1 cells, an adeno-associated virus-transformed human bronchial epithelial cell line derived from a CF patient (ΔF508/W1282X) and obtained from LGC Promochem, were grown as described previously [39]. Cells were infected with *P*. *aeruginosa* strains at a multiplicity of infection (MOI) of 100. Cell viability was evaluated using the CellTiter 96^®^ Non-Radioactive Cell Proliferation Assay (MTT) kit (Promega, G4000), according to manufacturer’s instructions.

### Bacterial isolates analysis

Bacterial isolates were plated on 1.5% BHI-agar plates and grown overnight at 37°C. Culture samples were taken and processed for genomic DNA and total RNA extraction. PAO1 and PA14 strains treated in the same conditions were used as controls. Oligos 9/10 and 37/38 were used for PCR-amplification of the genomic region containing the *pesA* and 16S (as positive PCR-control) *loci*, respectively.

## Results

### The sRNA PesA is encoded in the PAPI-1 and is a processed transcript

**Fig 1:**
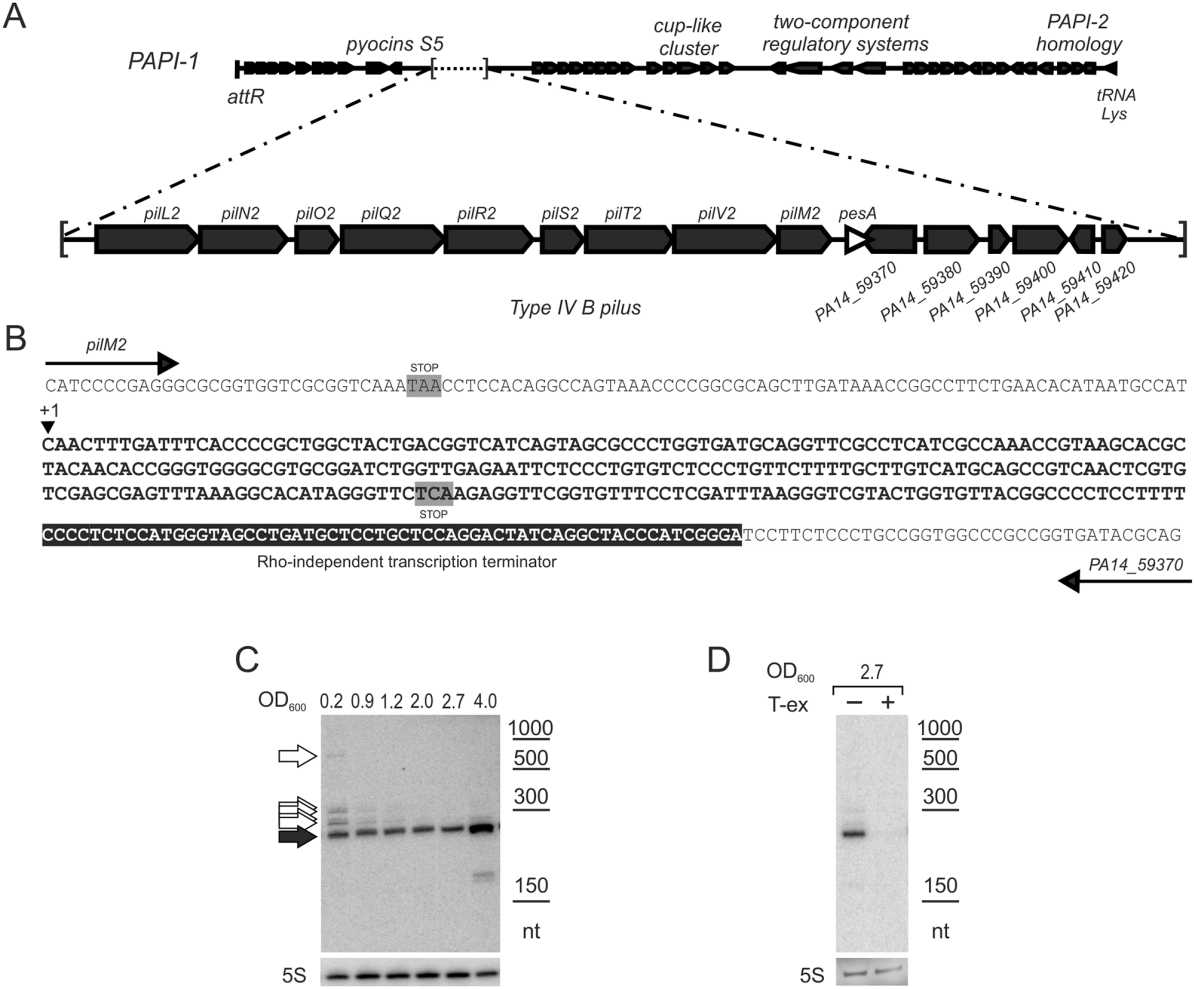
Genomic context and transcriptional features of *P*. *aeruginosa pesA* gene. A) Schematic overview of the PAPI-1 region of PA14. The *pesA* gene is indicated as white arrowhead. B) Sequence of the pilM2–PA14_59370 intergenic region of PA14. The *pesA* sequence is in bold. The 5′-end of PesA is indicated with +1. A predicted Rho-independent transcription terminator is highlighted in black. The predicted stop codons of pilM2 and PA14_59370 are highlighted in gray. C) PesA expression is induced in stationary phase. Wild-type PA14 was inoculated in BHI at an OD_600_ of 0.2 and grown for 20 h at 37°C with agitation. At the indicated OD_600_, culture samples were taken and processed for total RNA extraction and analysis by Northern blot. Black arrow indicates the main band of *pesA* of ≈ 260 nt; white arrows indicate bands of higher molecular weight of *pesA* present especially at early exponential phase (OD_600_ = 0.2). D) Northern blot analysis of PesA on 10 μg of total RNA extracted at the end of the exponential growth phase, treated (+) or untreated (−) with terminator 5′-phosphate-dependent exonuclease (T-ex).

The SPA0021 sRNA was identified using a comparative sRNA-seq approach in which 52 *P*. *aeruginosa* novel sRNAs have been identified either in the attenuated strain PAO1 or in the virulent one PA14. SPA0021 was validated to be ≈ 260 nt in lengths, and one of the 12 sRNAs whose genetic *locus* is unique to the PA14 strain [5]. Moreover, SPA0021 was found to be encoded within the pathogenicity island PAPI-1, and because of this property, we renamed it as pathogenicity island-encoded sRNA A (PesA). The *pesA* gene locates downstream and on the same strand of *pilM2* gene (belonging to the *type IV B pilus* operon *pil2*), and overlaps the 3’ of PA14_59370 (gene with unknown function) (Fig 1A and 1B). Since a rho-independent transcription terminator was predicted within the PA14_59370 sequence [40]. The cluster of the sRNA-seq reads that mapped upstream the predicted terminator was considered as the 3’ of the *pesA* gene [5]. The 5’-end was also mapped by the sRNA-seq read-clustering and was in perfect agreement with the size validated by Northern blot [5].

We evaluated PesA expression along the growth-curve in the rich medium BHI (Fig 1C). Northern blot analyses showed a main band of the expected product size of ≈ 260 nt (black arrow) at each analyzed time point, and multiple bands with higher molecular weight (white arrows) especially at early exponential phase (OD_600_ = 0.2). The main band of PesA showed to accumulate in late stationary phase. The presence of high molecular weight multiple bands, and the absence of rho-independent transcription terminators downstream *pilM2*, suggested that the main band of ≈ 260 nt could derive from the processing of a longer transcript. Sensitivity to treatment with terminator 5′-phosphate-dependent exonuclease (Fig 1D), which preferentially degrades monophosphate processed transcripts confirmed that the ≈ 260 nt RNA is indeed a processed product.

### PesA is widespread expressed in clinical and environmental isolates

To assess the clinical impact of PesA, we validated its presence and expression levels throughout a collection of 29 clinical *P*. *aeruginosa* isolates derived from respiratory samples of patients with chronic respiratory diseases, including CF and chronic obstructive pulmonary disease (COPD), and 5 isolates from environmental habitats. In particular, the clinical isolates were recovered both during intermittent infections and at different stages of chronic lung infection. Part of them was previously characterized both *in vitro* and *in vivo* [31, 41]. We also included in this study the Liverpool epidemic strain LESB58 [42] and PAO1 and PA14 as controls.

**Fig 2:**
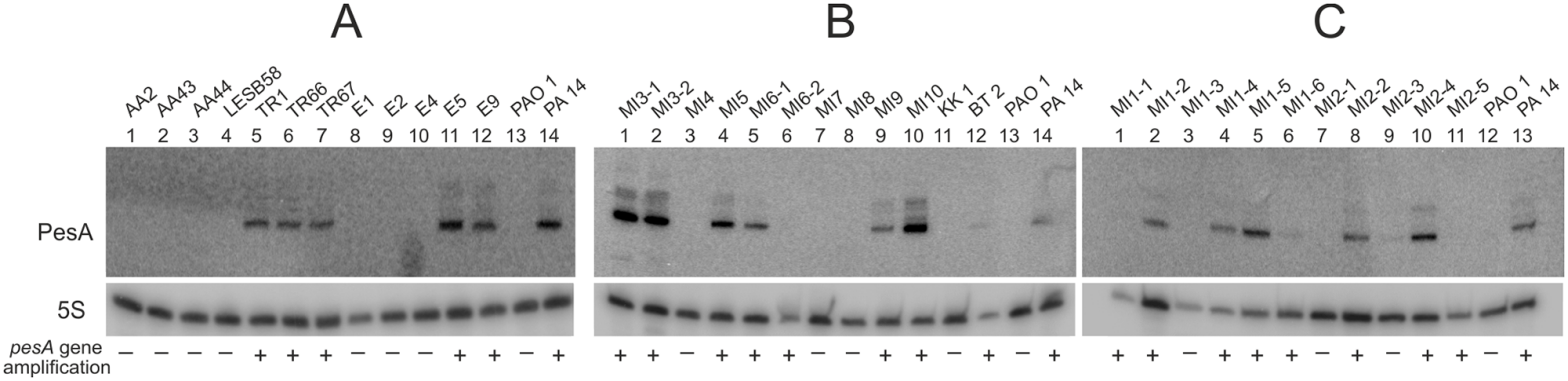
PesA gene dissemination and expression levels among environmental, CF and COPD clinical isolates. Assays on environmental, CF and COPD isolates are shown in three panels, A, B and C, respectively. The strain-collection was plated on BHI-agar plates. After overnight growth at 37°C, culture samples were taken and processed for total RNA extraction and analysis by Northern blot, and for genomic DNA extraction. Positive or negative PCR-amplification outcomes are indicated as “+” or “-” in the “*pesA* gene amplification” row, below each Northern Blot. PAO1 and PA14 were used as controls of Northern Blot analysis and for negative or positive gene amplification, respectively.

In Fig 2, results on *pesA*-gene amplification and Northern blot analysis show that the *pesA* gene is present in 17 out of the 27 CF clinical isolates, in the 2 COPD isolates and in 2 out of 5 environmental isolates. Notably, there was no association between the presence/expression of *pesA* gene and the *P*. *aeruginosa* status (chronic *vs* intermittent). In addition, no differences were observed between clonal isolates recovered from the same patients at different stages of chronic infection (e.g. AA2-early, AA43-late, AA44-late or TR1-early, TR66-late, TR67-late). In the BHI-plate aerobic growth conditions of these experiments, the majority of the *pesA*-harboring isolates showed levels of expression similar to those of the PA14 strain or even higher (panel A, lanes 5–7, 11 and 12; panel B, lanes 1, 2, 4, 5, 9, 10; panel C, lanes 2–5, 8 and 10), while 5 isolates showed lower or no expression levels with respect to the PA14 (panel B, lanes 6 and 12; panel C, lanes 1, 6 and 11). In the case of PAO1 and LESB58 strains, no gene amplification was observed (panel A, lanes 4 and 13).

### PesA is induced in anaerobic growth and at 37°C

**Fig 3:**
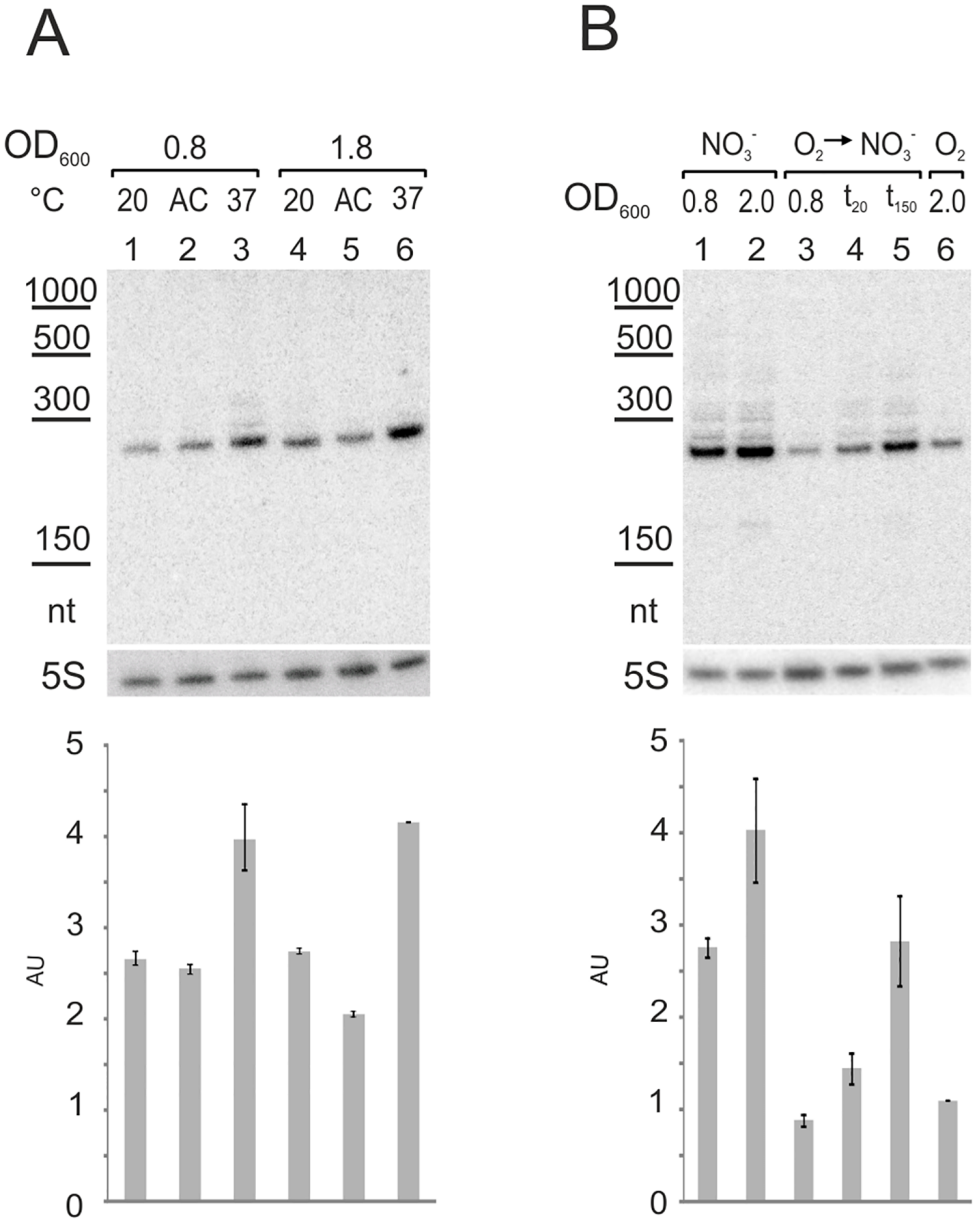
PesA expression is induced by temperature and low availability of oxygen. Levels of PesA RNA in: A) Wild-type PA14 grown in BHI at 20°C (lanes 1 and 4), 37°C (lanes 3 and 6) or following 20 min of acclimation (AC) from 20 to 37°C (lanes 2 and 5). Culture samples were taken at middle (OD_600_ of 0.8) and late (OD_600_ of 1.8) exponential growth phase. B) Wild-type PA14 grown in BHI anaerobically (NO_3_^−^; lanes 1 and 2), aerobically (O_2_, lane 6) and aerobically until an OD_600_ of 0.8 and then shifted to anaerobic conditions (O_2_ → NO_3_^−^; lanes 3, 4 and 5). Samples were taken 20 and 150 min after the shift to anaerobic conditions (t_20_ and t_150_). After sampling, cell cultures were processed for total RNA extraction and analysis by Northern blot. Intensities of the bands of PesA were quantified and normalized to those of 5S RNA in the same lane. Values are expressed as arbitrary units (AU) in the histograms below each Northern blot and represent the mean ± Standard Deviation (SD) of three independent experiments.

We analyzed the transcriptional responsiveness of PesA RNA to environmental or body temperature, and reduced oxygen availability. Temperature sensitivity was tested by probing PesA in early- (OD_600_ = 0.8) and mid-exponential phase (OD_600_ = 1.8) at both 20 and 37°C and after 20 min of acclimation following a shift from 20 to 37°C, as described in detail previously [17]. As shown in Fig 3A, the growth at 37°C caused an up-regulation of PesA if compared to the growth at 20°C; no increase of PesA RNA accumulation was observed during the 20 min of acclimation. PesA showed also to be responsive to oxygen availability. PesA was probed at mid- and late-exponential phase (OD_600_ of 0.8 and 2, respectively) under anaerobic conditions in BHI with nitrate to sustain anaerobic respiration, as described previously [17]. In addition, bacterial cells were grown in BHI with aeration until mid-exponential phase (OD_600_ = 0.8); then, oxygen was excluded from cultures. PesA levels were assessed immediately before oxygen exclusion and 20 (OD_600_ = 0.9) and 150 min (OD_600_ = 1.3) from the start of anaerobic conditions, as described previously [17]. PesA levels were higher in anaerobic than aerobic conditions both in mid- and late-exponential phase (Fig 3B). In addition, the shift from aerobic to anaerobic conditions caused a progressive increase of PesA levels.

### PesA is involved in ciprofloxacin and UV-resistance

To explore the involvement of PesA in the regulation of cellular mechanisms, also linked to *P*. *aeruginosa* virulence, we constructed the knock-out mutant strain PA14 *ΔpesA* and the plasmid vector pGM-*pesA* carrying the *pesA* gene under the arabinose inducible *P*_*BAD*_ promoter with the aim to measure the effects of perturbing PesA levels on phenotypic traits of the PA14 strain. Deletion of the *pesA* gene and overexpression from the pGM-*pesA* vector in the wild-type background were ensured by Northern blot (S1 Fig).

**Fig 4:**
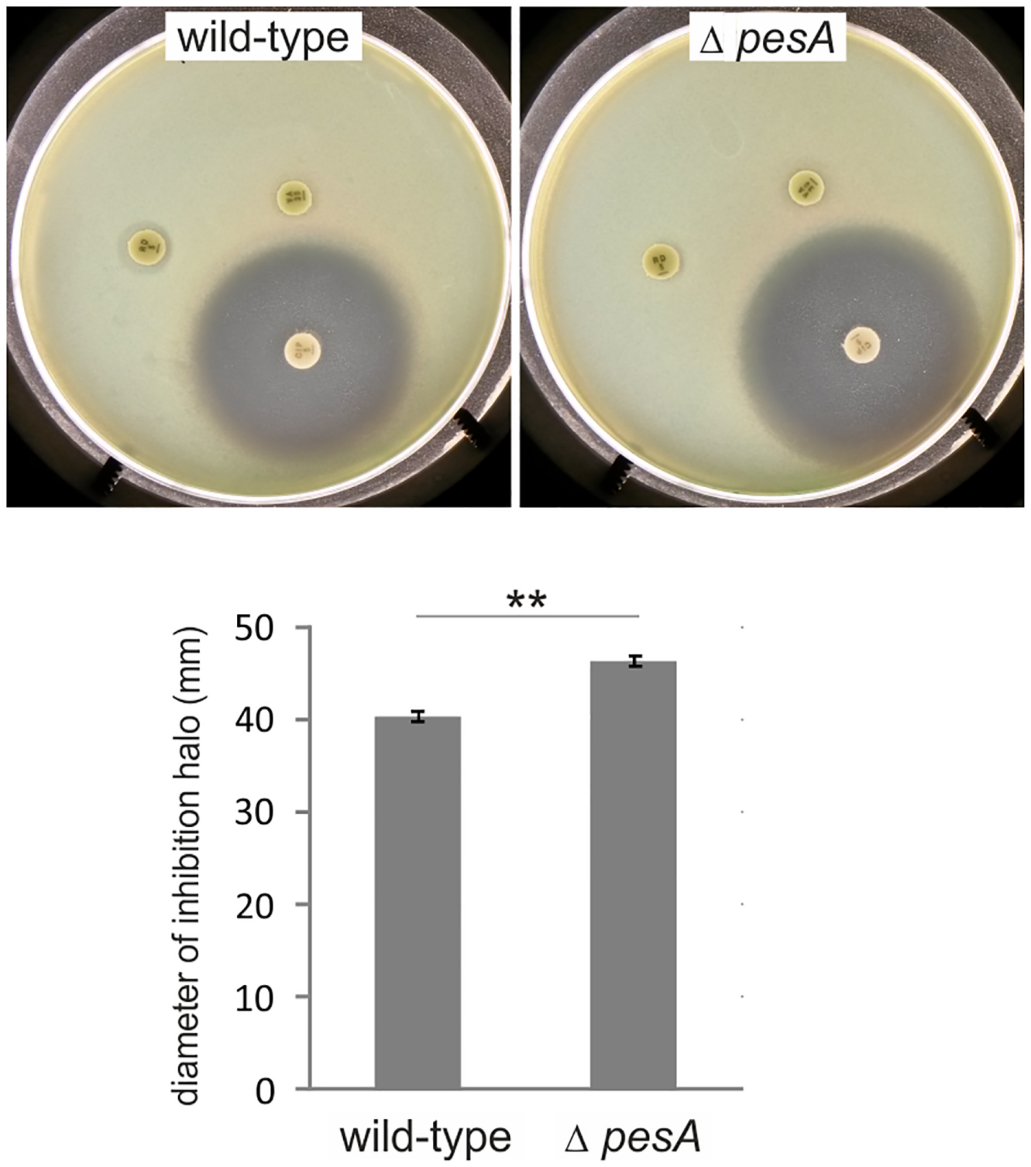
*PesA* deletion enhances sensitivity to ciprofloxacin. Antibiotic disk diffusion was performed on LB-agar plates spread with 10^6^ CFU bacterial cells of wild-type PA14 and *ΔpesA* mutant strains. The diameters of the inhibitory zones were measured after overnight incubation at 37°C. Data derive from three independent experiments. Values represent the mean ± SD. Statistical significance by Student’s t-Test is indicated: **p< 0.01.

**Fig 5:**
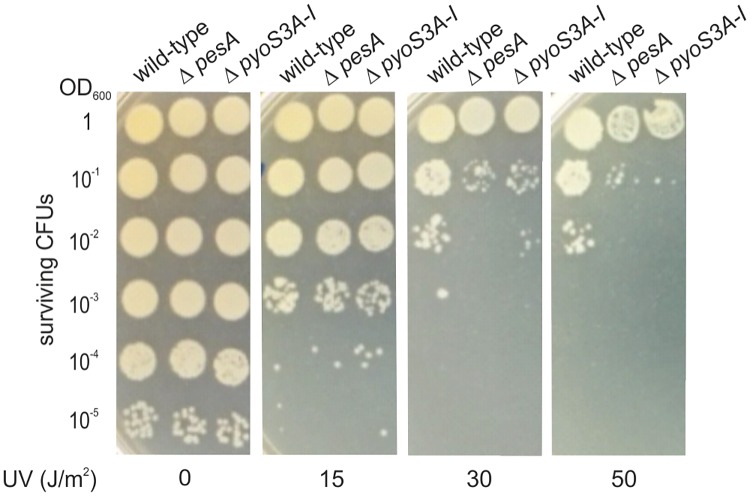
PesA deletion enhances UV sensitivity similarly to *pyoS3* operon deletion. 3 μl of cultures of PA14 *wt*, Δ*pesA and* Δ*pyoS3*, serially diluted 10-fold, were spotted onto LB-agar plates, and treated with UV light at the indicated doses. Surviving CFUs were observed after overnight incubation at 37°C.

We performed different types of phenotype evaluations. The most evident effects of PesA deletion were on UV and ciprofloxacin susceptibility. In particular, PesA deletion resulted in an enhanced sensitivity to the antibiotic ciprofloxacin and to UV irradiation. In fact, the susceptibility of wild-type PA14 and *ΔpesA* mutant cells to antimicrobials was analyzed by antibiotic disk diffusion on agar plates. The diameters of the inhibitory zones were measured after overnight incubation at 37°C. As shown in Fig 4, the diameter of the clear zone around ciprofloxacin was higher for mutant *ΔpesA* (46.33 ±0.58 mm) in comparison to that of wild-type strain (40.33 ± 0.58 mm) thus suggesting a contribution of PesA in the ciprofloxacin resistance mechanism. In addition, we noticed an incremented sensitivity of the *ΔpesA* mutant strain to UV light, showing a decrease in CFUs with respect to the wild-type starting from treatment with 30 J/m^2^ (Fig 5). This suggested the involvement of PesA in improving survival under conditions of genotoxic stress, such as UV irradiation treatment. Intriguingly, the deletion of *pyoS3A-I* operon, that is positively regulated by PesA (see below), gave rise to same levels of UV sensitivity as *pesA* deletion (Fig 5).

We did not observe significant differences for other phenotypes analyzed, including growth-curves analysis on rich and minimal media, susceptibility to other antimicrobial agents of different structural families, hemolytic activity, flagellum-mediated motility, pyocyanin and pyoverdine secretion.

### PesA is involved in *P*. *aeruginosa* pathogenicity in human CF respiratory cells

**Fig 6:**
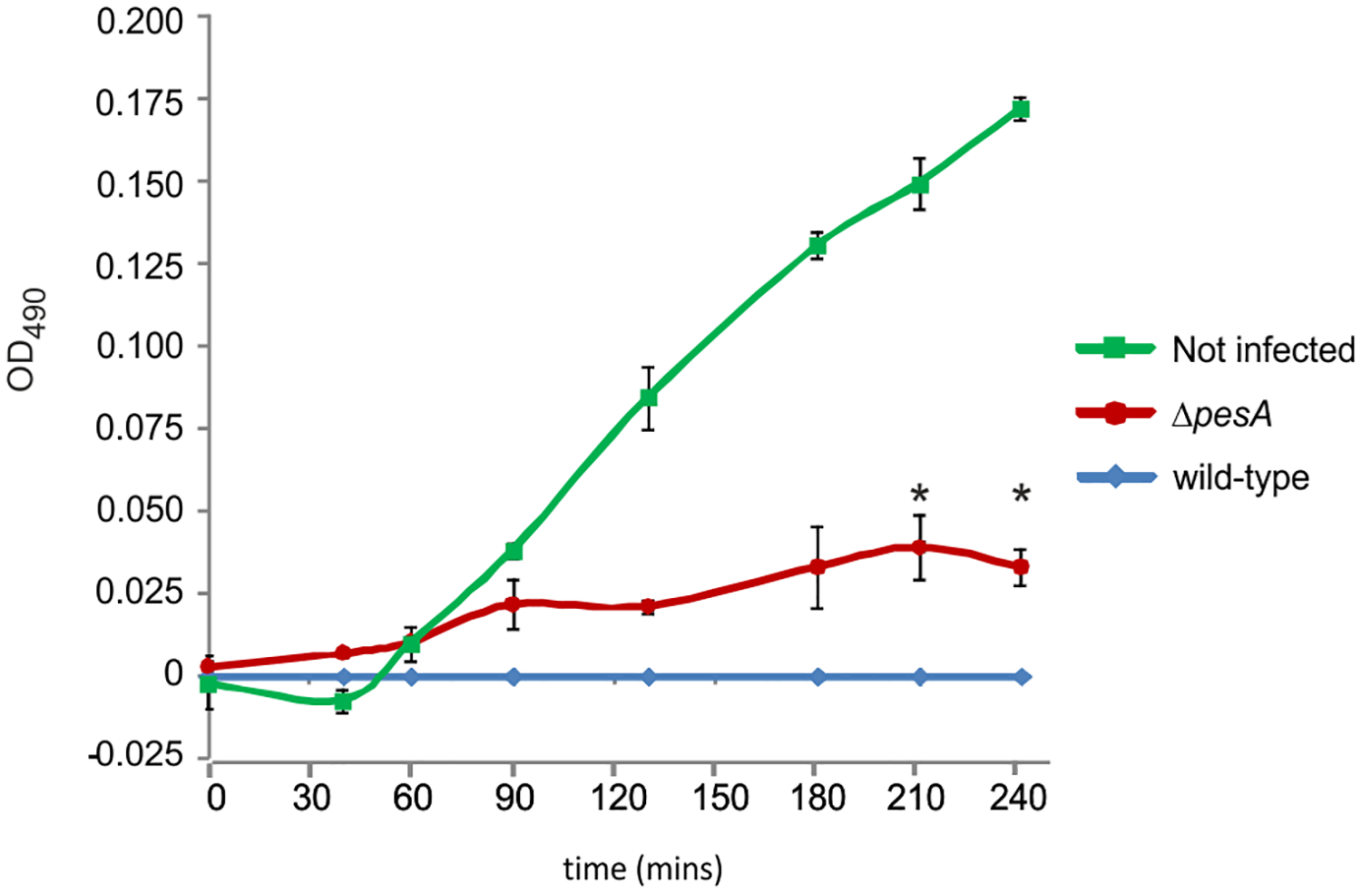
Time course of cell viability of IB3-1 cells following bacterial infection with *P*. *aeruginosa* PA14 wild-type and Δ*pesA*. Cell viability, assessed as a reduction of MTT salt, was quantified by the optical density (OD) at 490 nm. IB3-1 cells were seeded at a density of 5 × 10^4^ cells/well into 96-well microplates, and infected with 5 × 10^6^ bacterial cells (MOI 1:100). At every time point, data are shown as the difference in OD_490_ between the PA14 wild-type strain and the sRNA-deleted mutant Δ*pesA*. Uninfected cells were used as positive control of cell viability. Data derive from three independent experiments. Results are shown as the difference in the OD_490_ reached at the different time points by IB3-1 cells infected by the mutant strain or non-infected, subtracted of the OD_490_ reached by IB3-cells infected with the wild-type strain. Values represent the mean ± standard error of the mean (SEM). Statistical significance between wild-type and Δ*pesA* strains by Student’s t-Test is indicated: *p< 0.05.

We evaluated the virulence of *P*. *aeruginosa* PA14 strains on the CF bronchial epithelial cell line IB3-1. In particular, we assessed the killing capacity of PA14 Δ*pesA* mutant compared to the wild-type strain by the MTT assay, which provides a method of determining viable cell number measuring the conversion of 3-(4,5-dimethylthiazol)-2,5-diphenyltetrazolium bromide (MTT) to insoluble formazan by dehydrogenase enzymes of the intact mitochondria of living cells. Our results (Fig 6) showed that cells infected with *P*. *aeruginosa* PA14 Δ*pesA* mutant were more viable with respect to those infected by the wild-type strain, thus indicating that *pesA* may contribute to *P*. *aeruginosa* PA14 acute virulence.

### PesA targets *pyoS3A-I* operon

**Fig 7:**
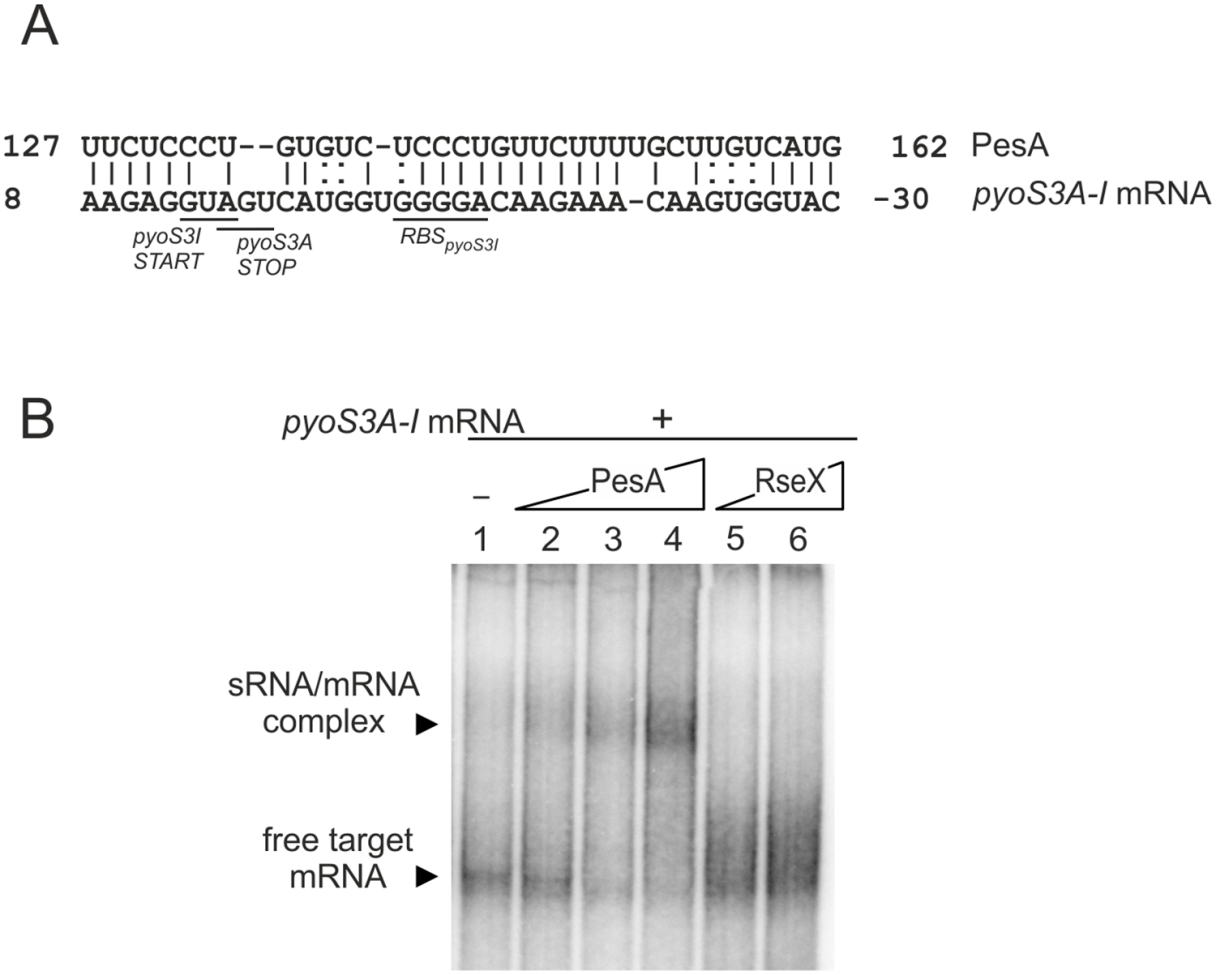
Interaction of PesA with *pyoS3A-I* mRNA. A) Prediction by *TargetRNA* software of the base-pairing interactions between PesA and *pyoS3A-I* mRNA. B) *In vitro* interaction between PesA RNA and *pyoS3A-I* mRNA by an electrophoretic mobility shift assay. Increasing amounts of PesA RNA (0, 0.08, 0.15, and 0.25 pmol; lanes 1–4) or, as a negative control, *E*. *coli* RseX RNA (0.25 and 2.5 pmol; lanes 5 and 6) were incubated at 37°C for 20 min with 0.15 pmol radiolabeled *pyoS3A-I* mRNA and loaded onto a native 6% polyacrylamide gel.

**Fig 8:**
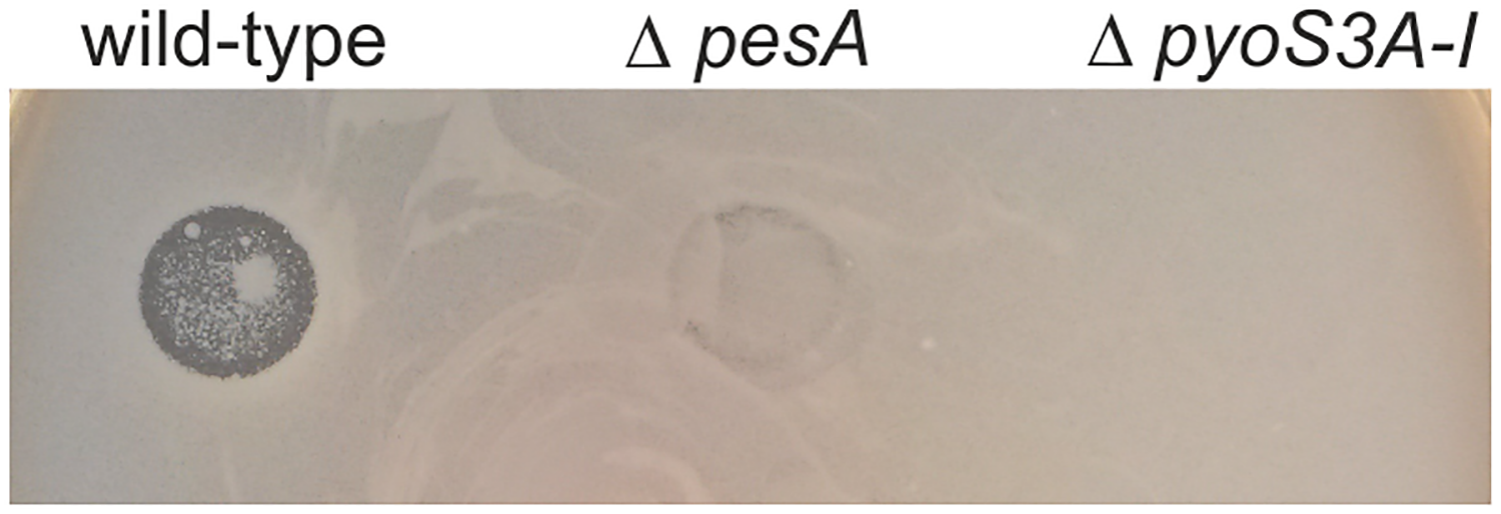
PesA deletion impairs the production of pyocin S3. Plate showing the effects of pyocin S3 present in the filtered supernatants of PA14 wild-type, Δ*pesA* and Δ*pyoS3A-I* on the killing of the indicator strain *P*. *aeruginosa* ATCC 27853. Drops of 5 μl of filtered supernatants from PA14 wild-type, Δ*pesA* and Δ*pyoS3A-I* cultures at OD_600_ = 1 were deposited on Luria-Bertani agar plates. A layer of the indicator strain *P*. *aeruginosa* ATCC 27853 was plated over the dried drops by inclusion in 0.7% agar. Plates were incubated overnight at 37°C. Clearer haloes represent inhibition (killing) of the indicator strain by pyocin S3 present in the sedimented supernatants.

As mentioned previously, PesA is encoded *in cis* to the 3’ of the gene PA14_59370 with unknown function, which makes the study of such putative target difficult to perform. Therefore, we managed to identify direct targets of PesA RNA by the use of the bioinformatics tool *TargetRNA* [43]. This tool predicted, as a high-scored output, an interaction in the region from -30 to -8 nt upstream the gene *pyoS3I* of the *pyoS3* operon (Fig 7A and S4 Table), predicted to encode pyocin S3 in the PA14 strain. The pyocin S3 genetic *locus* comprises two structural genes, *pyoS3A* (PA14_49520) and *pyoS3I* (PA14_49510), annotated to encode the killing S3A and the immunity S3I proteins, respectively. To confirm this annotation in PA14, we deleted the *pyoS3* operon and tested the pyocin S3 production by the killing assay of the sensitive ATCC 27853 *P*. *aeruginosa* strain [27]. As shown in Fig 8, deletion of *pyoS3A-I* completely abolished the production of pyocin S3 by PA14 strain. Remarkably, PesA deletion resulted in strong reduction of pyocin S3 production, thus suggesting a positive role of PesA on the pyocin S3 production.

The predicted region of the *pyoS3A-I* operon targeted by PesA comprises the Ribosome Binding Site (RBS) of the *pyoS3I* gene, and locates within the ORF of the *pyoS3A* gene. To assess this predicted PesA-*pyoS3* mRNA interaction, PesA RNA and the *pyoS3* mRNA region spanning –116 to +108 from *pyoS3I* translational start site were produced *in vitro*, mixed and analyzed on native polyacrylamide gels. As shown in Fig 7B, the two RNAs specifically formed a complex.

**Fig 9:**
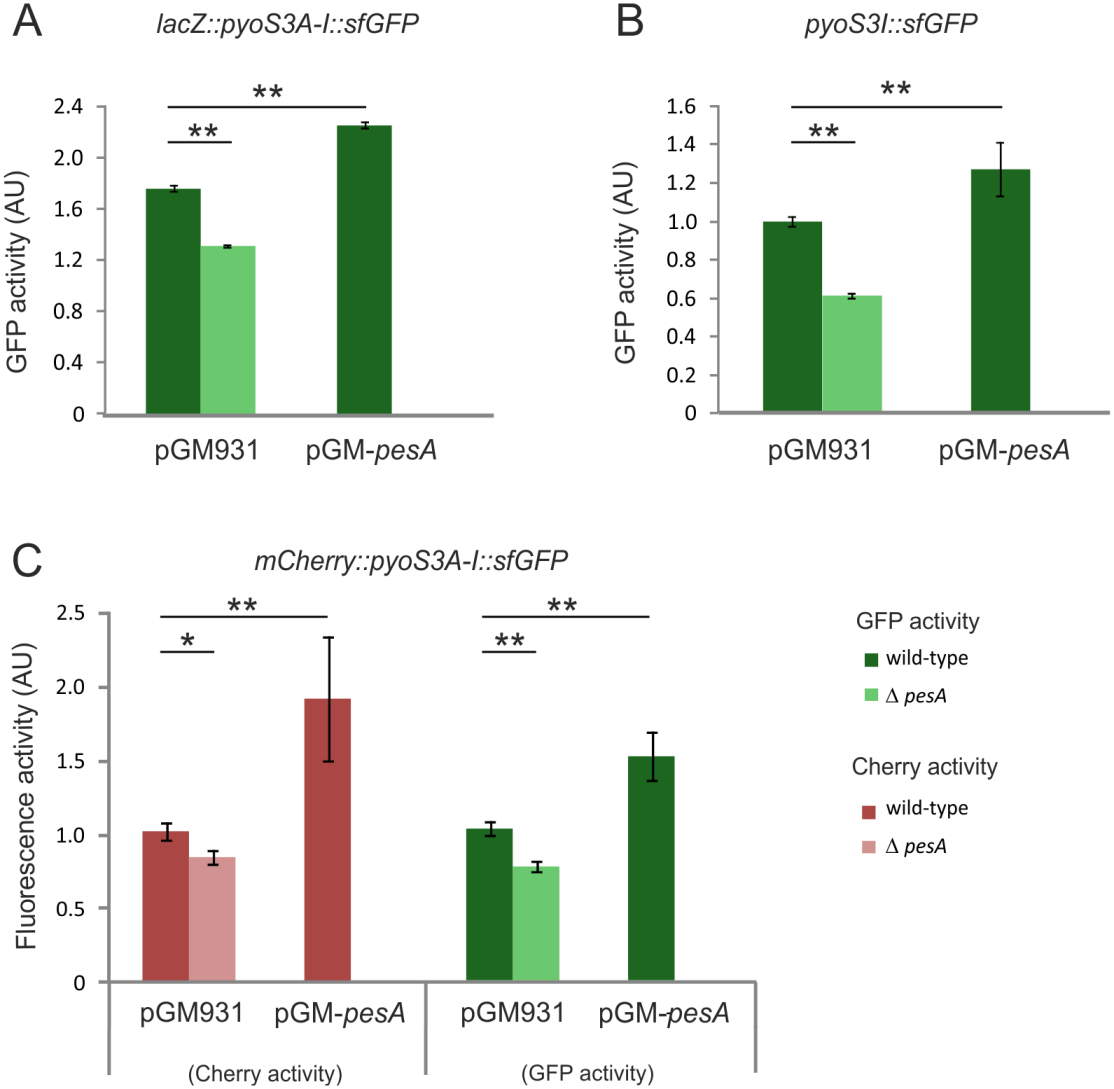
PesA positively regulates the expression of both the *pyoS3A* and *pyoS3I* translational fusions in PA14. Comparison of the sfGFP and mCherry activities expressed in arbitrary units (AU) resulting from the translational fusion of (A) *lacZ*::*pyos3A-I*::*sfGFP*, (B) *pyoS3I*::*sfGFP* and (C) *Cherry*::*pyoS3A-I*::*sfGFP* combined with the control vector (pGM931) or the plasmid overexpressing PesA (pGM-*pesA*), in PA14 wild-type and PA14 Δ*pesA*. The strains were grown to an OD_600_ of 1.8 in LB medium supplemented with gentamicin and carbenicillin, to maintain pBBR1- and pGM- plasmids, respectively, and arabinose, to induce PesA overexpression. Cells were harvested and treated for sfGFP and mCherry activity determination by measuring fluorescence polarization FP_485/535_ and fluorescence intensity FI_590/635_, respectively. sfGFP and mCherry activities are expressed as ratio FP_485/535_/Abs_595_ and FI_590/635_/Abs_595_, respectively. Data derive from three independent experiments. Values represent the mean ± SD. Statistical significance by Student’s t-Test is indicated: *p<0.05; **p< 0.01.

We generated distinct types of translational fusions to test the effects of PesA on the *pyoS3I* gene alone and on the *pyoS3A-I* mRNA. A first reporter plasmid, named pBBR1-*lacZ*::*pyoS3A-I*::*sfGFP*, mimics a bi-cistron under the control of the heterologous constitutive promoter *P*_*LtetO-1*_. It was obtained by cloning a region of 224 nt, comprehensive of the last 117 nt of the *pyos3A* gene and the first 108 nt of *pyoS3I* thus generating a first translational fusion of the reporter F’*lacZ* with the last 39 codons of *pyoS3A*, and a second translational fusion of the first 36 codons of *pyoS3I* gene with *sf*GFP. GFP activity was assayed in PA14 wild-type and PA14 *ΔpesA* in the absence and presence of PesA overexpression from pGM-*pesA*. As shown in Fig 9A, there was an approximately 25% reduction in GFP activity in the PA14 *ΔpesA* background. In the presence of pGM-*pesA* overexpressing PesA, in wild-type background, GFP activity increased approximately 30%. These results suggested that PesA positively regulates *pyoS3I* expression. To test whether translation of *pyos3A* gene was necessary to have this PesA effects on *pyos3I*, we generated a second mono-cistronic reporter derivative, pBBR1-*pyoS3I*::*sfGFP*, carrying the same *pyoS3A-I* region as pBBR1-*lacZ*::*pyoS3A-I*::*sfGFP* in which only the *pyoS3I* gene was translationally fused with sfGFP. GFP analyses confirmed that PesA influence in a positive manner the regulation of *pyoS3I* in the mono-cistronic construct, with a ~20% increment in GFP activity in the presence of PesA overexpression in the wild-type background, and a ~40% decrease in the PA14 *ΔpesA* background (Fig 9B). This suggested that the translation of the two genes is not merely and solely coupled and that the effect of PesA on *pyoS3I* do not require the translation of *pyoS3A*.

To assess the influence of PesA also on the *pyoS3A* gene, we substituted the sequence coding for the F’LacZ domain in pBBR1-*lacZ*::*pyoS3A-I*::*sfGFP* with the one of the reporter gene *mCherry*, and monitored simultaneously the activity of both mCherry and sfGFP of the translational fusion *mCherry*::*pyoS3A*-*S3I*::*sfGFP*. As shown in Fig 9C, the ~50% increase in sfGFP activity followed by PesA overexpression, and the ~25% decrease in the PA14 *ΔpesA* background reconfirmed the positive regulation exerted by PesA on the *pyoS3I* gene. mCherry also showed an increase in activity when PesA was overexpressed, suggesting that PesA also exerts a positive regulation on the upstream gene *pyoS3A*. Thus, PesA seems to be a positive regulator of the whole *pyoS3* operon.

Quantitative RT-PCR on total mRNA of wild-type and Δ*pesA* strains was also performed to check whether PesA influenced *pyoS3* mRNA levels. Samples were taken at mid-, late-exponential and stationary phase (OD_600_ of 0.8, 1.6 and 2.7, respectively) and both genes, *pyoS3A* and *pyoS3I*, were analyzed for their expression levels. No significant differences were observed either for *pyoS3A* or *pyoS3I* in wild-type and Δ*pesA* backgrounds at any time-point. Notably, the expression levels of the two genes were comparable at every time-point and seemed not to be influenced by the growth phase, being constant along the growth curve (S5 Table). We conclude that PesA exerts positive regulation on *pyoS3A-I* mRNA by modulating mRNA translatability and without influencing its stability.

By the use of the *IntaRNA* web tool [44, 45], we also detected a putative interacting region between PesA and the leader sequence of the *pyoS3* operon, from -76 to -42 from the TTG start codon of the *pyoS3A* gene. To evaluate whether PesA was also able to influence the S3-operon translation by acting on its leader sequence, we generated the translational fusion *leader-pyoS3A*::*sfGFP*, by cloning the whole leader region of the *pyoS3* operon and the sequence encoding the first 37 aminoacids of *pyos3A* gene, in frame with the *sfGFP* reporter. The comparison of the fluorescence activity of the *leader-pyoS3A*::*sfGFP* translational fusion between the wild-type and Δ*pesA* genetic background did not show any significant difference, not even in presence of PesA overexpression from pGM-*pesA* in wild-type (S2 Fig). Spurious outside interactions of PesA with sfGFP and mCherry open reading frame were ruled out using alternative reporter plasmids carrying the *sfGFP* and *mCherry* genes alone (S2 Fig).

## Discussion

We studied the novel *P*. *aeruginosa* sRNA PesA, which was originally identified as being transcribed from the horizontally acquired pathogenicity island PAPI-1 in the strain PA14. Our analysis revealed that PesA is widespread in clinical isolates from patients affected by chronic respiratory diseases, such as CF, being expressed in 55% of the cases tested. Moreover, PesA expression is responsive to low oxygen conditions, a hallmark of CF and COPD, and impacts *P*. *aeruginosa* pathogenicity in CF bronchial cells. These results suggest that PesA could be relevant during *P*. *aeruginosa* infection in chronic respiratory diseases.

We speculated that PesA had the potential to act as a *trans*-encoded base-pairing sRNA involved also in the post-transcriptional regulation of genes located outside PAPI-1 and thus performed genome-wide bioinformatics screenings for target genes. One predicted target was the *pyoS3A-I* operon coding for pyocin S3. We then confirmed that both *pyoS3A* and *pyoS3I* genes are positively influenced by PesA. The interaction of PesA with the bicistronic *pyoS3A-I* mRNA is suggested to simultaneously stimulate translation initiation and termination of *pyoS3I* and *pyoS3A*, respectively, without influencing stability of the *pyoS3A-I* mRNA. Interestingly, PesA could impact a putative mechanism of translation coupling between *pyoS3I* and *pyoS3A* [22] that remains to be elucidated. However, in this paper we demonstrate that stimulation of *pyoS3I* by PesA does not require translation of the upstream *pyoS3A* thus suggesting that the two genes are not strictly translationally coupled. This scenario, as a whole, is compatible with the role of PesA in assuring balanced expression of toxin S3A and antitoxin S3I to prevent deleterious effects on the producing host. Overall, these data obtained with the reporter genes are consistent with the observation that PesA deletion results in strong reduction of pyocin S3 production (Fig 8).

It is conceivable that PesA can be involved in mechanisms of niche establishment via pyocin S3. PesA was in fact also detected in environmental isolates and its expression from PAPI-1 might confer a selective advantage that favours PAPI-1 maintenance in the environment due to its regulation of pyocin S3.

It is likely that PesA has a broad set of target genes whose functions go beyond the niche establishment. The observation that PesA deletion induces less killing in infected CF bronchial epithelial cells suggests that PesA could modulate, directly or indirectly, virulence factors of *P*. *aeruginosa*. It was previously shown that pyocin S2 is endowed with cytotoxic activity on human cell lines [46]. It can be argued that pyocin S3 has similar effects and can act as virulence factor whose regulation is under the control of PesA.

Furthermore, PesA could regulate the expression of genes involved in DNA damage repair as suggested by the increased sensitivity of *ΔpesA* mutant to fluoroquinolone antibiotic ciprofloxacin and to UV irradiation. Intriguingly, the degree of UV sensitivity displayed by the *ΔpesA* mutant is comparable to that of a strain deleted for *pyoS3A*-*I*. These results imply a potential involvement of the DNase activity of pyocin S3 in DNA damage repair, and introduce an intriguing network among sRNAs, pyocins and DNA damage repair that will require additional experiments to be elucidated. To our knowledge this is the first work characterizing a sRNA encoded in a pathogenicity island in *P*. *aeruginosa*. In addition, our results indicate that PesA is able to modulate key genes located outside the PAPI-1. In summary, the horizontal acquisition of PAPI-1 could provide the new host with a regulatory function that can switch the expression of genes involved in niche establishment, virulence and stress resistance.

## Supporting information

S1 Fig Validation of PesA deletion and overexpression. A) PA14 wild-type and PA14 Δ*pesA* were grown in BHI medium until an OD_600_ of 2.7. Culture samples were taken and processed for total RNA extraction and analysis by Northern blot. B) PA14 strains harbouring pGM-*pesA* or the control empty vector pGM931 were grown in BHI medium with carbenicillin until an OD_600_ of 0.8. Cells were split into two flasks, and 10 mM arabinose (ara) was added to one. Culture samples were taken at the indicated OD_600_ and processed for total RNA extraction and analysis by Northern blot probing PesA RNA. Intensities of the bands of PesA were quantified and normalized to those of 5S RNA in the same lane. Values are expressed as arbitrary units (AU) in the histograms below the Northern blot.
(TIF)

S2 Fig Fluorescence activity to check PesA regulation on the leader of *pyoS3A* gene, and spurious outside interactions of PesA with *sfGFP* and *mCherry* open reading frames. A) Comparison of the sfGFP activity resulting from the translational fusion *leader-pyoS3A*::*sfGFP* in PA14 wild-type and PA14 Δ*pesA* (-), and combined with the control vector (pGM931) or the plasmid overexpressing PesA (pGM-*pesA*) in PA14 wild-type. B) Comparison of the fluorescence activity of the reporter gene *sfGFP* combined with the control vector (pGM931) or the plasmid overexpressing PesA (pGM-*pesA*), in PA14 wild-type and PA14 Δ*pesA*. C) Comparison of fluorescence activity of the reporter gene *mCherry* combined with the control vector (pGM931) or the plasmid overexpressing PesA (pGM-*pesA*), PA14 wild-type and PA14 Δ*pesA*.
(TIF)

S1 Table Clinical isolates. (PDF)

S2 Table Strains and plasmids. (PDF)

S3 Table Oligonucleotides. (PDF)

S4 Table List of mRNA targets of PesA predicted by bioinformatics analysis conducted with the *TargetRNA* web-tool. (PDF)

S5 Table Quantitative RT-PCR analyses of *pyoS3A* and *pyoS3I* mRNA levels in wild-type and Δ*pesA* backgrounds. (PDF)
