# Supplementary figures and images for: The PAPI-1 pathogenicity island-encoded small RNA PesA influences Pseudomonas aeruginosa virulence and modulates pyocin S3 production
Source: PLoS One. 2017 Jun 30;12(6):e0180386. doi: 10.1371/journal.pone.0180386 (PMC5493400; doi:10.1371/journal.pone.0180386)

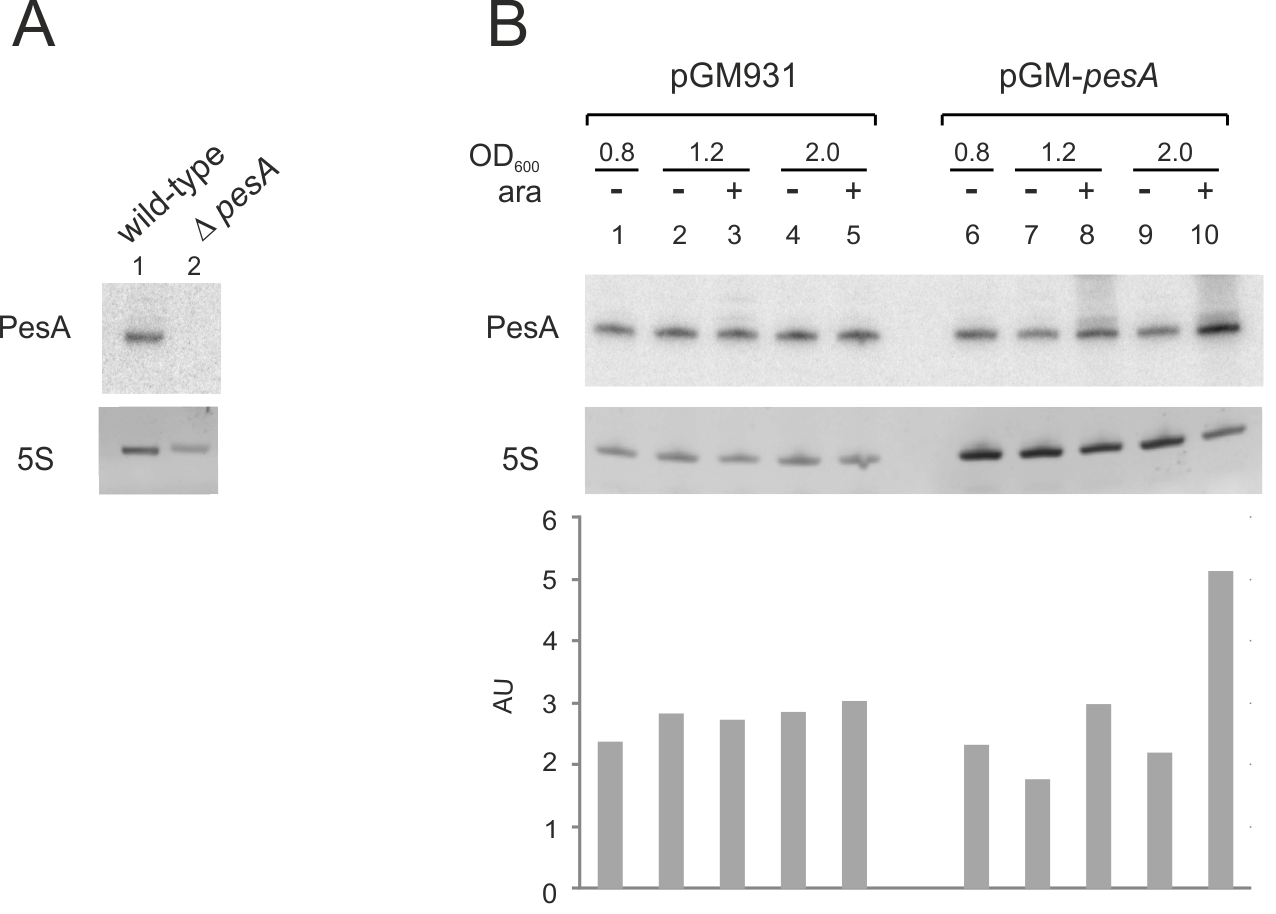

Supplement: S1 Fig — A) PA14 wild-type and PA14 ΔpesA were grown in BHI medium until an OD600 of 2.7. Culture samples were taken and processed for total RNA extraction and analysis by Northern blot. B) PA14 strains harbouring pGM-pesA or the control empty vector pGM931 were grown in BHI medium with carbenicillin until an OD600 of 0.8. Cells were split into two flasks, and 10 mM arabinose (ara) was added to one. Culture samples were taken at the indicated OD600 and processed for total RNA extraction and analysis by Northern blot probing PesA RNA. Intensities of the bands of PesA were quantified and normalized to those of 5S RNA in the same lane. Values are expressed as arbitrary units (AU) in the histograms below the Northern blot. (TIF) [file pone.0180386.s001.tif]

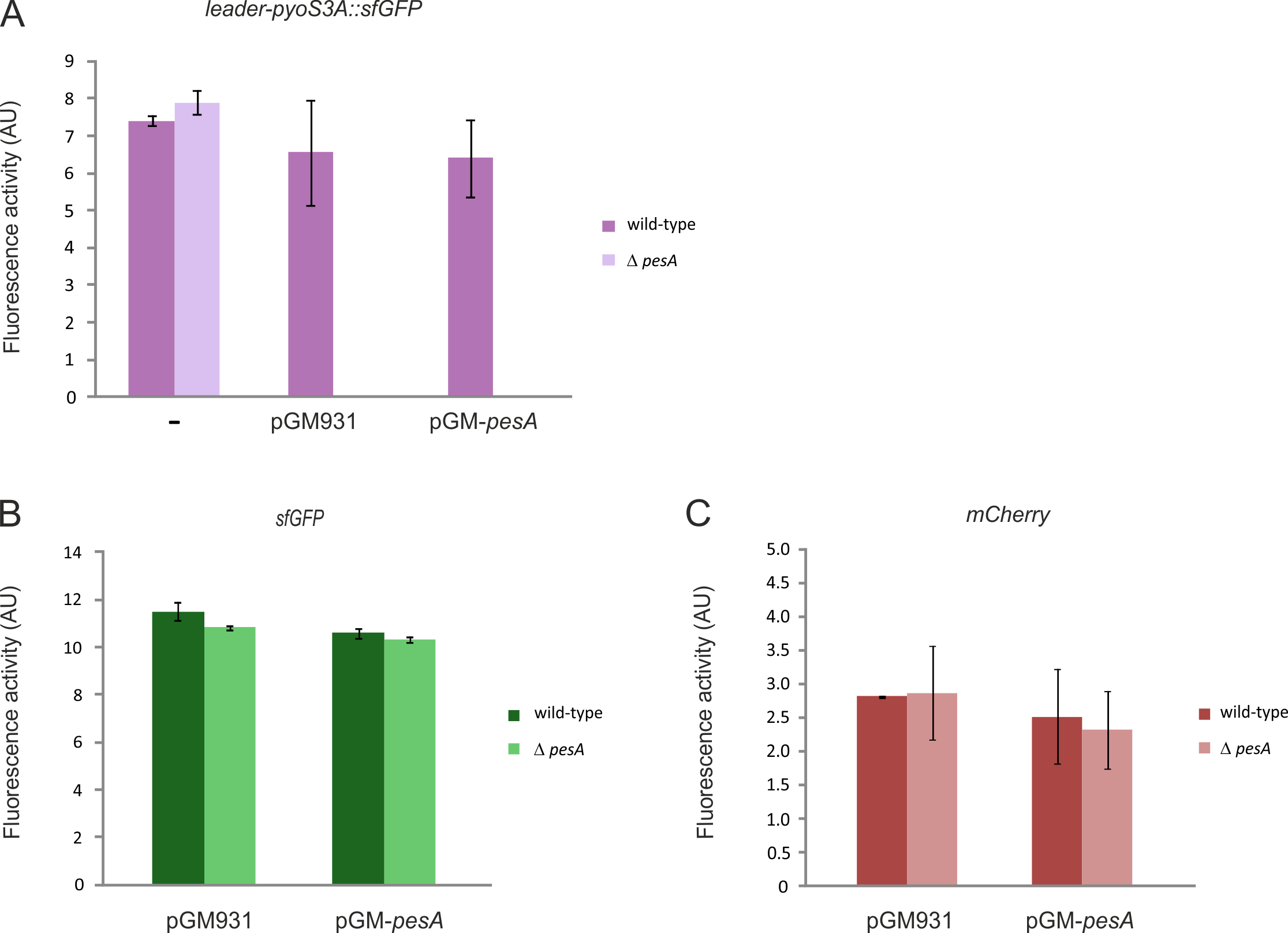

Supplement: S2 Fig — A) Comparison of the sfGFP activity resulting from the translational fusion leader-pyoS3A::sfGFP in PA14 wild-type and PA14 ΔpesA (-), and combined with the control vector (pGM931) or the plasmid overexpressing PesA (pGM-pesA) in PA14 wild-type. B) Comparison of the fluorescence activity of the reporter gene sfGFP combined with the control vector (pGM931) or the plasmid overexpressing PesA (pGM-pesA), in PA14 wild-type and PA14 ΔpesA. C) Comparison of fluorescence activity of the reporter gene mCherry combined with the control vector (pGM931) or the plasmid overexpressing PesA (pGM-pesA), PA14 wild-type and PA14 ΔpesA. (TIF) [file pone.0180386.s002.tif]
