# Supplementary material for: The PAPI-1 pathogenicity island-encoded small RNA PesA influences Pseudomonas aeruginosa virulence and modulates pyocin S3 production
Source: PLoS One. 2017 Jun 30;12(6):e0180386. doi: 10.1371/journal.pone.0180386 (PMC5493400; doi:10.1371/journal.pone.0180386)
Supplement: S1 Table — (PDF) [file pone.0180386.s003.pdf]

S1 Table. Clinical isolates

Patient	Disease[a]	Sex	CFTR mutation	*P. aeruginosa* Isolate	*P. aeruginosa* status	Range of age (years)
MI3	CF	M	F508del/F508del	MI3-1	Intermittent	16-20
				MI3-2	Intermittent	16-20
MI4	CF	M	F508del/1717-1G-->A	MI4	Intermittent	26-30
MI5	COPD	F	-	MI5	Intermittent	21-25
MI6	CF	F	UNK/UNK	MI6-1	Chronic	41-45
				MI6-2	Chronic	41-45
MI7	CF	M	G542X/R1066H	MI7	Chronic	21-25
MI8	CF	M	F508del/2183-AA-->G	MI8	Chronic	16-20
MI9	CF	F	F508del/F508del	MI9	Chronic	21-25
MI10	COPD	M	-	MI10	Intermittent	1-5
MI1	CF	F	UNK/UNK	MI1-1	Chronic	41-45
				MI1-2	Chronic	41-45
				MI1-3	Chronic	41-45
				MI1-4	Chronic	41-45
				MI1-5	Chronic	41-45
				MI1-6	Chronic	41-45
MI2	CF	M	F508del/F508del	MI2-1	Intermittent	1-5
				MI2-2	Intermittent	1-5
				MI2-3	Intermittent	1-5
				MI2-4	Chronic	6-10
				MI2-5	Chronic	6-10

[a] CF: Cystic Fibrosis; COPD: Chronic Obstructive Pulmonary Disease.
