# Supplementary material for: The PAPI-1 pathogenicity island-encoded small RNA PesA influences Pseudomonas aeruginosa virulence and modulates pyocin S3 production
Source: PLoS One. 2017 Jun 30;12(6):e0180386. doi: 10.1371/journal.pone.0180386 (PMC5493400; doi:10.1371/journal.pone.0180386)
Supplement: S2 Table — (PDF) [file pone.0180386.s004.pdf]

Table S2. Strains and plasmids

Strains or Plasmids	Genotype/Relevant features	Construction	Reference
Pseudomonas aeruginosa			
PA14	wild-type		[1]
PA14 Δ*pesA*	markerless Δ*pesA*		this work
PAO1	wild-type		[2]
ATCC 27853			[3]
Escherichia coli			
C1a	*E. coli C*, prototrophic		[4]
TOP10	*mcrA* Δ(*mrr-hsd*RMS-*mcr*BC) Φ80*lac*ZΔM15 Δ *lac*X74 *rec*A1 *ara*D139 Δ(*araleu*)7697 *gal*U *gal*K *rps*L (StrR) *end*A1 *nup*G		Invitrogen
CC118 *λpir*	Δ(*ara-leu*), *araD*, Δ*lac*X74, *galE*, *galK*, *phoA*, *thi1*, *rpsE*, *rpoB*, *argE* (Am), *recA1*, lysogenic (*λpir*)		[5]
Plasmids			
pHERD20T	*araBp*- t_Ω, P_{BAD}, Ap^r		[6]
pGM931	pHERD20T derivative, araC/P_{BAD} - t_Ω, Ap^r		[7]
pGM-*pesA*	pGM931 derivative, *pesA* under P_{BAD}, Ap^r	contains PCR product using oligo 9/10	this work
pXG10-SF	sfGFP reporter plasmid; *lacZ*::*sfGFP* under $P_{LtetO\text{-}1}$, Cm^r		[8]
pXG30-SF	sfGFP reporter plasmid; *FlacZ'*::*sfGFP* under $P_{LtetO\text{-}1}$, Cm^r		[8]
pMMR	mCherry reporter plasmid, Tc^r		[9]
pXG10-*pyoS3I::sfGFP*	pXG10-SF derivative; $P_{LtetO\text{-}1}$ -->*pyoS3I::sfGFP*, Cm^r	contains PCR product using oligo 11/12	this work
pXG30-*pyoS3A-I::sfGFP*	pXG30-SF derivative; $P_{LtetO\text{-}1}$ --> *FlacZ'::pyoS3A-I::sfGFP*, Cm^r	contains PCR product using oligo 11/12	this work
pXG10-*mCherry::pyoS3A-I::sfGFP*	pXG10-*pyoS3I::sfGFP* AND pMMR derivative ; $P_{LtetO\text{-}1}$ --> *mCherry::pyoS3A-I::sfGFP*, Cm^r	contains PCR product using oligo 15/16	this work
pXG10-*leader-pyoS3A::sfGFP*	pXG10-SF derivative; $P_{LtetO\text{-}1}$ -->*pyoS3A::sfGFP*, Cm^r	contains PCR product using oligo 13/14	this work
pBBR1-MCS5	REP, lacZ alpha, Gm^r		[10]
pBBR1-*sfGFP*	sfGFP reporter plasmid; $P_{LtetO\text{-}1}$ --> *sfGFP*, Gm^r		[11]
pBBR1-*mCherry*	pBBR1-MCS5 and pXG10-*mCherry::pyoS3A-I::sfGFP* derivative, $P_{LtetO\text{-}1}$ --> *mCherry*, Gm^r	contains PCR product using oligo 17/18	this work

pBBR1-*pyoS3I::sfGFP*	pBBR1-MCS5 and pXG10-*pyoS3I::sfGFP* derivative, $P_{LtetO-1}$ --> *FLacZ'::pyoS3A-I::sfGFP*, Gm^r	contains PCR product using oligo 18/19	this work
pBBR1-*lacZ::pyoS3A-I::sfGFP*	pBBR1-MCS5 and pXG30-*pyoS3A-I::sfGFP* derivative; $P_{LtetO-1}$ --> *FLacZ'::pyoS3A-I::sfGFP*, Gm^r	contains PCR product using oligo 18/19	this work
pBBR1-*mCherry::pyoS3A-I::sfGFP*	pBBR1-MCS5 and pXG10-*mCherry::pyoS3A-I::sfGFP* derivative, $P_{LtetO-1}$ --> *mCherry::pyoS3A-I::sfGFP*, Gm^r	contains PCR product using oligo 18/19	this work
pBBR1-*leader-pyoS3A:: sfGFP*	pBBR1-MCS5 and pXG10-*leader-pyoS3A::sfGFP* derivative, $P_{LtetO-1}$ -->*pyoS3A::sfGFP*, Gm^r	contains PCR product using oligo 18/19	this work
pSEVA612S	OriR6K, lacZαMCS flanked by two I-*Sce*I, Gm^r, Ap^r		[12]
pSW-1	OriRK2, XylS, Pm-->I-*Sce*I, Ap^r		[13]
pSEVApa14-Δ*pesA*	pSEVA612S derivative, Ap^r	contains SOEing-PCR product of the PA14-amplificates TS1 and TS2 using oligo 23/24 and 25/26	this work

References

1. Rahme LG, Stevens EJ, Wolfort SF, Shao J, Tompkins RG, Ausubel FM. Common virulence factors for bacterial pathogenicity in plants and animals. Science. 1995;268(5219):1899-902.

2. Stover CK, Pham XQ, Erwin AL, Mizoguchi SD, Warrener P, Hickey MJ, et al. Complete genome sequence of Pseudomonas aeruginosa PAO1, an opportunistic pathogen. Nature. 2000;406(6799):959-64.

3. Baysse C, Meyer JM, Plesiat P, Geoffroy V, Michel-Briand Y, Cornelis P. Uptake of pyocin S3 occurs through the outer membrane ferripyoverdine type II receptor of Pseudomonas aeruginosa. J Bacteriol. 1999;181(12):3849-51.

4. Sasaki I, Bertani G. Growth abnormalities in Hfr derivatives of Escherichia coli strain C. J Gen Microbiol. 1965;40(3):365-76.

5. de Lorenzo V, Timmis KN. Analysis and construction of stable phenotypes in gram-negative bacteria with Tn5- and Tn10-derived minitransposons. Methods Enzymol. 1994;235:386-405.

6. Qiu D, Damron FH, Mima T, Schweizer HP, Yu HD. PBAD-based shuttle vectors for functional analysis of toxic and highly regulated genes in Pseudomonas and Burkholderia spp. and other bacteria. Appl Environ Microbiol. 2008;74(23):7422-6.

7. Delvillani F, Sciandrone B, Peano C, Petiti L, Berens C, Georgi C, et al. Tet-Trap, a genetic approach to the identification of bacterial RNA thermometers: application to Pseudomonas aeruginosa. RNA. 2014;20(12):1963-76.

8. Corcoran CP, Podkaminski D, Papenfort K, Urban JH, Hinton JC, Vogel J. Superfolder GFP reporters validate diverse new mRNA targets of the classic porin regulator, MicF RNA. Mol Microbiol. 2012;84(3):428-45.

9. Popat R, Crusz SA, Messina M, Williams P, West SA, Diggle SP. Quorum-sensing and cheating in bacterial biofilms. Proc Biol Sci. 2012;279(1748):4765-71.

10. Kovach ME, Eschbach EJ, Wheeler DL. Quantification methodology for peripheral quantitative computed tomography (PQCT) data using public domain software. Biomed Sci Instrum. 2004;40:161-7.

11. Ferrara S, Carloni S, Fulco R, Falcone M, Macchi R, Bertoni G. Post-transcriptional regulation of the virulence-associated enzyme AlgC by the sigma(22) -dependent small RNA ErsA of Pseudomonas aeruginosa. Environ Microbiol. 2015;17(1):199-214.

12. Martinez-Garcia E, de Lorenzo V. Engineering multiple genomic deletions in Gram-negative bacteria: analysis of the multi-resistant antibiotic profile of Pseudomonas putida KT2440. Environ Microbiol. 2011;13(10):2702-16.

13. Wong SM, Mekalanos JJ. Genetic footprinting with mariner-based transposition in Pseudomonas aeruginosa. Proc Natl Acad Sci U S A. 2000;97(18):10191-6.
