# Supplementary material for: The PAPI-1 pathogenicity island-encoded small RNA PesA influences Pseudomonas aeruginosa virulence and modulates pyocin S3 production
Source: PLoS One. 2017 Jun 30;12(6):e0180386. doi: 10.1371/journal.pone.0180386 (PMC5493400; doi:10.1371/journal.pone.0180386)
Supplement: S3 Table — (PDF) [file pone.0180386.s005.pdf]

S3 Table. Oligonucleotides

Oligo #	Oligo Name	Sequence (5' → 3') [a]	Utilization
1	SPA0021	GGAGGGGCCGTAACACCAGTAC	Northern-blot probe for PesA
2	PA5SRNA02	GGAGACCCCACACTACCATCGGCGATG	Northern-blot probe for 5S
3	T7_5'spa0021_sense_f	**CTAATACGACTCACTATAGGG**CAACTTTGATTTCACCC	Amplification of PesA in fusion with RNA polymerase T7 promoter for *in vitro* transcription
4	3'spa0021_sense_r	AAAAGGAGGGGCCGTAACACCAGTACG	
5	T7_ -116ATG_pyoS3I_sense_ f	**CTAATACGACTCACTATAGGG**GATAGCAATTTCGATGCC	Amplification of the *pyoS3A-I* region spanning -116 to +108 from PyoS3I translational start site, in fusion with RNA polymerase T7 promoter for *in vitro* transcription
6	pyoS3I +108ATG_r	TTCGTAGGGGCTCTCCAG	
7	T7_RseX_f	**CTAATACGACTCACTATAGGG**TTTTTATTATTCTGTGTCATGATG	Amplification of RseX in fusion with RNA polymerase T7 promoter for *in vitro* transcription
8	RseX_r	TAAAAAAAAGCCGGCATCAT	
9	NcoI_5'SPA0021_f	GAccatggCAACTTTGATTTCACCCCG	Amplification of PesA with *Nco*I/*Pst*I ends for cloning in pGM931 vector, and for verification of *pesA* gene in bacterial isolates
10	PstI_3'SPA0021_r	GAActgcagAAAAGGAGGGGCCGTAACA	
11	-116ATG_ pyoS3I_NsiI_f	GTTTTatgcatGATAGCAATTTCGATGCCGTCAAG	Amplification of the *pyoS3A-I* region spanning -116 to +108 from *pyoS3I* translational start site with *Nsi*I/*Nhe*I ends for cloning in frame with *sfGFP* and/or *FlacZ'* in pXG10-SF and pXG30-SF vectors
12	pyoS3I_+108ATG_NheI_r	GTTTTgctagcTTCGTAGGGGCTCTCCAG	
13	PyoS3A_leader_NsiI_f	GTTTTatgcatAAAACGCTGGGCTCGCAATCA	Amplification of the 5' UTR and beginning of *pyoS3A* with *Nsi*I/*Nhe*I ends for cloning in frame with *sfGFP* in pXG10-SF vector
14	PyoS3A_+114nt ATG_NheI_r	GTTTTgctagcTGAGTATCTAACAATCAGTGGC	
15	mCherry_SD_NsiI_f	GTTTTatgcatCACAGGAGGAACAGCATGGTGAGCAAGGGCGAG	Amplification of *mCherry* reporter gene with *Nsi*I ends for cloning in frame with *pyoS3A* in pXG10-*pyoS3I::sfGFP* vector
16	mCherry_Fus_NsiI_r	GTTTTatgcatCTTGTACAGCTCGTCCATGCCGCC	

17	mCherry_TAA_ XbaI_r	GTTTTtctaga**TTA**CTTGTACAGCTCGTCCAT		Amplification from pXG10-*mCherry::pyoS3A-I::sfGFP* construct, from the $P_{LtetO-1}$ promoter to the codon before the last of *mCherry* and added with the stop codon, with *Cla*I/*Xba*I ends for cloning in pBBR1-MCS5
18	Ptet-O1_ClaI_f	GTTTTatcgatTCCCTATCAGTGATAGAG	Amplification from the $P_{LtetO-1}$ promoter to the stop codon of *sfGFP* of pXG10- and pXG30- derivatives with *Cla*I/*Xba*I ends for cloning in pBBR1-MCS5	
19	sfGFP_TAA_ XbaI_r	TGATGCCtctagaTTATTTGTAGAGCTC		
20	sfGFP_+96_r	TTGTGCCCATTAACATCACCATC	Reverse primer on *sfGFP* for verification of constructs	
21	pSEVA_f	TAAAACGACGGCCAGTATAGGG	Forward primer on pSEVA vector for verification of contructs/sequencing of TS1 and TS2	
22	pSEVA_r	CAGCTATGACCATGATTACGCC	Reverse primer on pSEVA vector for verification of contructs/sequencing of TS1 and TS2	
23	TS1_SPA0021_ EcoRI_f	GCgaattcTAGCAGGTCGCCACGACTCAA	Amplification of the genomic region TS1 using genomic DNA of PA14	
24	TS1_SPA0021_r	TGACAAGCAAAAGAACAGGGCGGCCGCTGCGCCGGGGTTTACTGGCCTGT		
25	TS2_SPA0021_f	GCGGCCGCCCTGTTCTTTTGCTTGTCA	Amplification of the genomic region TS2 using genomic DNA of PA14	
26	TS2_SPA0021_ PstI_r	GAActgcagGATCACGCGTTCATCATCGAA		
27	TS1SPA0021_ 300up_f	AACGGGAAATGGGGACGAAC	Forward primer annealing 300 bp upstream the *pesA* gene for verification of deletion	
28	TS2SPA0021_ 300down_r	GGAACTCACGCACAACACTG	Reverse primer annealing 300 bp downstream the *pesA* gene for verification of deletion	
29	pHERD_rev	TGCAAGGCGATTAAGTTGGGT	Reverse primer on pHERD 20T/pGM931 for verification of constructs	
30	16S_f	TGTCGTCAGCTCGTGTCGTGA	Amplification of 16S in bacterial isolates and for real time-PCR analysis	
31	16S_r	ATCCCCACCTTCCTCCGGT		

32	pyoS3A_Fw_RT	GCAGAGTTGGATACGGAAGCTG	Amplification of *pyoS3A* for real time-PCR analysis
33	pyoS3A_Rv_RT	ATTCGTTATCTAGTTCAGGGCGCTT	
34	pyoS3I_Fw_RT	TCTGAACACCTAACGCTCTCTCC	Amplification of *pyoS3I* for real time-PCR analysis
35	pyoS3I_Rv_RT	CTAACTAGAGGTTCAGGCAGGCT	

[a] Lowercase: sites for restriction enzymes. Bold: T7 promoter sequence. Underlined: Shine-Dalgano sequence. Bold Underlined: stop codon
