# Supplementary material for: The PAPI-1 pathogenicity island-encoded small RNA PesA influences Pseudomonas aeruginosa virulence and modulates pyocin S3 production
Source: PLoS One. 2017 Jun 30;12(6):e0180386. doi: 10.1371/journal.pone.0180386 (PMC5493400; doi:10.1371/journal.pone.0180386)
Supplement: S4 Table — (PDF) [file pone.0180386.s006.pdf]

S4 Table. List of mRNA targets of PesA predicted by bioinformatics analysis conducted with the *TargetRNA* web-tool

PROGRAM PARAMETERS

Species:	*Pseudomonas_aeruginosa_PA14*
Remove terminator:	*yes*
Before Start Codon:	*-30*
After Start Codon:	*20*
Hybridization Seed:	*9*
G:U Pairs in Seed:	*no*
Single Target:	-
P-value:	*0.01*
Thermodynamic:	*no*
Orthologs:	-

	Gene	Synonym	Score	P-value	sRNA_start	sRNA_stop	mRNA_start	mRNA_stop
1	pyoS3I	PA14_49510	-78	0.0018243	127	162	-30	8
2	PA14_03330	PA14_03330	-77	0.0021381	158	186	-23	4
3	PA14_11520	PA14_11520	-77	0.0021381	19	62	-29	13
4	ppiD	PA14_41190	-75	0.00293667	140	172	-11	20
5	PA14_12990	PA14_12990	-74	0.00344148	90	115	-6	20
6	PA14_24740	PA14_24740	-74	0.00344148	136	170	-27	8
7	pilS	PA14_60250	-74	0.00344148	44	67	-30	-4
8	tesB	PA14_12870	-72	0.00472571	219	242	-28	-3
9	PA14_07200	PA14_07200	-71	0.0055372	233	262	-15	13
10	pqsH	PA14_30630	-71	0.0055372	20	51	-14	18
11	rnhA	PA14_41060	-71	0.0055372	142	163	-2	19
12	nosZ	PA14_20200	-69	0.0076004	154	204	-28	20
13	PA14_11050	PA14_11050	-68	0.00890341	183	209	-9	16
14	PA14_17000	PA14_17000	-68	0.00890341	147	162	-1	15
15	PA14_44800	PA14_44800	-68	0.00890341	56	73	-19	-2
16	PA14_52900	PA14_52900	-68	0.00890341	217	233	-16	1
17	PA14_63370	PA14_63370	-68	0.00890341	55	70	1	17
