# Supplementary material for: The PAPI-1 pathogenicity island-encoded small RNA PesA influences Pseudomonas aeruginosa virulence and modulates pyocin S3 production
Source: PLoS One. 2017 Jun 30;12(6):e0180386. doi: 10.1371/journal.pone.0180386 (PMC5493400; doi:10.1371/journal.pone.0180386)
Supplement: S5 Table — (PDF) [file pone.0180386.s007.pdf]

S5 Table. Quantitative RT-PCR analyses of *pyoS3A* and *pyoS3I* mRNA levels in wild-type and Δ*pesA* backgrounds

Strain	OD_{600}	***pyoS3A***	***pyoS3I***
PA14 wt	0.8	1	1
PA14 Δ*pesA*	0.8	1.39	1.24
PA14 wt	1.6	1.19	1.26
PA14 Δ*pesA*	1.6	1.21	1.47
PA14 wt	2.7	1.08	1.15
PA14 Δ*pesA*	2.7	1.66	1.48

Relative expression of the *pyoS3A* and *pyoS3I* genes determined by quantitative Real-Time PCR on total RNA extracted from PA14 wild-type (wt) Δ*pesA*. Samples were taken at OD_{600} of 0.8, 1.6 and 2.7. 16S RNA transcript was used as reference gene. *Δ*Ct values between the genes of interest and 16S RNA were set at 1 for PA14 wt OD_{600} 0.8.
